# An Analysis of Human MicroRNA and Disease Associations

**DOI:** 10.1371/journal.pone.0003420

**Published:** 2008-10-15

**Authors:** Ming Lu, Qipeng Zhang, Min Deng, Jing Miao, Yanhong Guo, Wei Gao, Qinghua Cui

**Affiliations:** 1 Department of Medical Informatics, Peking University Health Science Center, Beijing, China; 2 Ministry of Education Key Laboratory of Molecular Cardiology, Peking University, Beijing, China; 3 Department of Pediatrics, Peking University First Hospital, Beijing, China; Center for Genomic Regulation, Spain

## Abstract

It has been reported that increasingly microRNAs are associated with diseases. However, the patterns among the microRNA-disease associations remain largely unclear. In this study, in order to dissect the patterns of microRNA-disease associations, we performed a comprehensive analysis to the human microRNA-disease association data, which is manually collected from publications. We built a human microRNA associated disease network. Interestingly, microRNAs tend to show similar or different dysfunctional evidences for the similar or different disease clusters, respectively. A negative correlation between the tissue-specificity of a microRNA and the number of diseases it associated was uncovered. Furthermore, we observed an association between microRNA conservation and disease. Finally, we uncovered that microRNAs associated with the same disease tend to emerge as predefined microRNA groups. These findings can not only provide help in understanding the associations between microRNAs and human diseases but also suggest a new way to identify novel disease-associated microRNAs.

## Introduction

MicroRNAs (miRNAs) are a class of small non-coding RNAs (∼22 nt) which normally function as negative regulators of target mRNA expression at the posttranscriptional level. They bind to the 3′UTR of target mRNAs through base pairing, resulting in target mRNAs cleavage or translation inhibition[1], [2], [3]. It has also recently been demonstrated that miRNAs may function as positive regulators in some cases[4], [5]. It is estimated that 1–4% genes in the human genome are miRNAs and a single miRNA can regulate as many as 200 mRNAs[6]. There is increasing evidence suggesting that miRNAs play critical roles in many key biological processes, such as cell growth, tissue differentiation, cell proliferation, embryonic development, and apoptosis[6]. We previously found that miRNA also play important roles in cellular signaling network[7], cross-species gene expression variation[8], and co-regulation with transcription factors[9]. As such, mutation of miRNAs, dysfunction of miRNA biogenesis and dysregulation of miRNAs and their targets may result in various diseases. Currently, there have been reported ∼70 diseases are associated with miRNAs (see our database http://cmbi.bjmu.edu.cn/hmdd.).

Many studies have produced a large number of miRNA-disease associations and shown that the mechanisms of miRNAs involved in diseases are very complex. In such a complex case, a comprehensive analysis of these data will do great help in understanding the associations between miRNAs and diseases. Furthermore, a large-scale analysis and integration of these miRNA-disease associations will offer a platform to dissect the patterns of the miRNA and disease associations, even though current known miRNA-disease associations are far from completeness. In this study, we reported our literature-based generation of miRNA-disease associations and the analysis of these data based on bioinformatics.

## Results

### The human miRNA disease database

We retrieved miRNA-disease associations from ∼100 papers and built a human miRNA-associated disease database (HMDD), which contains miRNA names, disease names, dysfunction evidences, and PubMed ID. HMDD is publicly accessible at website: http://cmbi.bjmu.edu.cn/hmdd. This database was built on November 2007 and the data at that time was used in the following analysis. The last update was made on July 2008. The miRNA-disease association data between November 2007 and July 2008 was used to validate the main results in this study.

### Dysfunctional evidences in the clusters of the miRNA-associated disease network

A bipartite graph can be used as a network model to connect two disjoint sets of nodes [10], [11]. As shown in Supplementary Figure S1, a bipartite graph contains two sets of nodes. And nodes in the same set are not connected and edges only exist between nodes from different sets. Here, we constructed a bipartite graph consisting of two disjoint sets of nodes based on the associations between a list of miRNAs and a list of human diseases obtained from HMDD. One set contains human miRNA-associated diseases (69 diseases) and the other set contains disease-associated miRNAs (238 miRNAs). Based on the human miRNA-disease bipartite graph, we constructed the miRNA-associated disease network (MDN) by giving two diseases an edge if they share at least one common associated miRNA. The MDN network shows cluster structures (Supplementary Figure S2), in which similar diseases are clustered together. All cancers are connected together (Supplementary Figure S2), suggesting that various cancers may share similar associations at the miRNA level, in which some strong onco-miRNAs or miRNA suppressors may play key roles. For example, miR-21 is overexpressed in various cancers from almost all studies, showing a feature of strong onco-miRNAs, whereas miR-125a shows down-regulation in various cancers, suggesting that it is a miRNA suppressor. Similarly, all cardiovascular diseases are also connected together, which may result from some cardiovascular disease related miRNAs, such as miR-1 and miR-133, which play roles in almost all cardiovascular diseases in the MDN. The cancer cluster is clearly separated from the cardiovascular disease cluster (Supplementary Figure S2). These two clusters are connected with each other through only several hub diseases, such as heart failure, cardiac hypertrophy, and skeletal muscle hypertrophy. One exception is neointimal hyperplasia, a cardiovascular disease, which is predominantly connected to the cancer cluster and has only two connections to other cardiovascular diseases. Thus, it may share more common miRNA associations with cancers than with cardiovascular diseases.

Diseases in the same cluster are highly interconnected; for example, each node connects to an average of 26 other cancers in the cancer cluster and 9 other cardiovascular diseases in the cardiovascular disease cluster, whereas cancers connect to only 5 cardiovascular diseases on average. Disease-associated miRNAs show various dysfunctions, such as mutation, up-regulation, deleted, and down-regulation. However, it remains unclear whether diseases sharing the same miRNAs have the same miRNA dysfunctions. Because most of the reported miRNA dysfunctions are either up-regulation or down-regulation, we curated most of the dysfunctions in the human miRNA disease database into these two groups. We assigned terms such as “deleted” and “low expressed” into the down-regulation group and terms such as “overexpressed”, “highly expressed”, and “over expression” into the up-regulation group. We next investigated the dysfunctional patterns within the same cluster as well as between different clusters. We calculated the number of the same dysfunctions (both up-regulation or both down-regulation) and the number of different dysfunctions (one shows up-regulation and the other shows down-regulation) for miRNAs that link diseases within the cancer cluster, or the cardiovascular disease cluster (intra-cluster), and between cancer and cardiovascular disease clusters (inter-cluster). Most of the shared miRNAs show the same dysfunctions in diseases in the same clusters, for example, 82% (578/706) of the disease pairs in the cancer cluster show the same dysfunctions and 77% (68/88) of the disease pairs in the cardiovascular disease cluster show the same dysfunctions (Table 1). However, only 54% (92/170) of the paired diseases between the cancer cluster and the cardiovascular disease cluster shows the same dysfunctions (Table 1). Diseases in different clusters thus show a larger fraction of different miRNA dysfunctions (P = 5.97×10^−13^, Fisher's Exact Test). For example, miR-195 is up regulated in all reported cardiovascular diseases and down regulated in all reported cancers. This result suggested that although different classes of diseases associated with common miRNA, the underlying association mechanisms might be different.

**Table 1:** The miRNA dysfunction pattern of diseases in the same clusters and between clusters.

Diseases pairs	In cluster1	In cluster2	Intra-cluster	Between-clusters
Num1	578	68	646	92
Num2	128	20	148	78
P value			P = 5.97×10^−13^	

Cluster 1 is the cancer cluster.

Cluster 2 is the cardiovascular disease cluster.

Num1 is the number of the same dysfunction evidences.

Num2 is the number of different dysfunction evidences.

P value was calculated using Fisher's Exact Test.

### miRNA tissue specificity and miRNA related diseases

**Figure 1:**
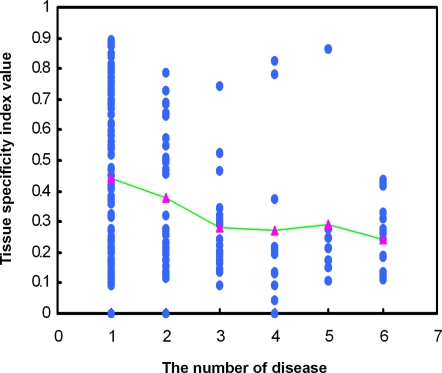
The association of miRNA tissue specificity and the number (Nd) of diseases it implicated in. Each blue circle represents a miRNA whose x,y coordinates are its Nd and its tissue specificity index value. MiRNAs are grouped into different groups according to the Nd, and then the average tissue specificity index value of each group was calculated, shown as pink triangle. The pink triangles are connected with green lines.

It has been reported that tissue-specific miRNAs are often implicated in diseases related to specific tissues[12], [13], [14]. However, it remains largely unknown whether there is a correlation between the tissue specificity of a miRNA and the number of diseases associated with it. In order to dissect this question, we first obtained the miRNA expression profiles of 345 miRNAs across 40 normal tissues from a recently published paper[15]. We then used the tissue specificity index τ to measure the tissue specificity of a miRNA[16]. The τ value ranges from 0 to 1. A higher τ value indicates higher tissue specificity of that miRNA. We next classified the human disease related miRNAs into several groups according to the number of diseases in which a miRNA is implicated. We then calculated the average tissue specificity index value for each group of miRNAs. Finally, we observed a negative correlation between the tissue specificity index and the number of diseases in which a miRNA is implicated (Figure 1, R = −0.83, P = 0.058, Spearman's correlation). Most of the miRNAs associated with a large number of diseases (> = 4) show low tissue specificity index values (Figure 1). However, there are a few outliers; miR-372, miR-373, and miR-206 that are associated with 5, 4, and 4 diseases, whereas they show high tissue specificity index values of 0.86, 0.82, and 0.78, respectively. miR-372 and miR-373 are specifically expressed in placenta, while miR-206 is specifically expressed in skeletal muscle. We also noted that some miRNAs associated with only one disease show low tissue specificity, which may mainly result from the incompleteness of the miRNA-disease associations, that is, currently only one disease was reported to associate with that miRNA. As the research going on, more diseases would be reported to associate with these low tissue specific miRNAs. This result revealed a potential correlation between miRNA tissue specificity and disease, which may be of value in predicting specific disease-related miRNAs by combining the miRNA tissue specificity values. Thus, if a disease occurs specifically in a given tissue, the miRNAs specifically expressed in that tissue will have a great potential to be related to that disease. However, most of the tissue specific miRNAs have not been reported related to diseases. Using this method, we detected miRNAs that show high tissue specificity (using τ> = 0.80 as a cutoff, Supplementary Text S1), which are potentially associated with that tissue-specific diseases. The dissection of these relationships will be valuable for studying the functions of these miRNAs and their mechanisms in diseases and can be used to discovering novel disease-associated miRNAs.

### miRNA conservation and miRNA related diseases

It has been reported that there exists a significant association between a gene's (protein's) connectivity and its conservation in yeast Saccharomyces cerevisiae [17] and human [18]. It is expected that if a gene is evolutionarily conserved it would have more connections to other genes and be more lethal when dysfunctions. Therefore, its dysfunction would result in diseases with a higher probability. However, it remains largely unknown whether this association exists for human miRNAs and diseases. To investigate this issue, we classified all the human disease-associated miRNAs into two groups: miRNAs that are conserved in other species (group 1) and miRNAs that are human specific (group 2). We assigned one human miRNA into the conserved miRNAs if its family members can be found in other species according to the family annotations of miRBase[19]. Otherwise, we assigned it into the human-specific group. We counted the number of miRNAs that are associated with at least one disease or zero disease for these two groups. Our results show that miRNAs in group 1 tend to be associated with diseases with a higher probability significantly (P = 3.9×10^−33^, Fisher's Exact Test, Table 2).

**Table 2:** The number of miRNAs implicated in one or more than one human diseases for two groups.

	Group1	Group2
Num1	191	128
Num2	213	1
P value	3.9×10^−33^	

Groups1 contains 404 human miRNAs conserved in other species.

Group2 contains 129 human specific miRNAs.

Num1 is the number of miRNAs that are not reported related to disease.

Num2 is the number of miRNAs implicated in at least one disease.

P value was calculated using Fisher's Exact Test.

**Figure 2:**
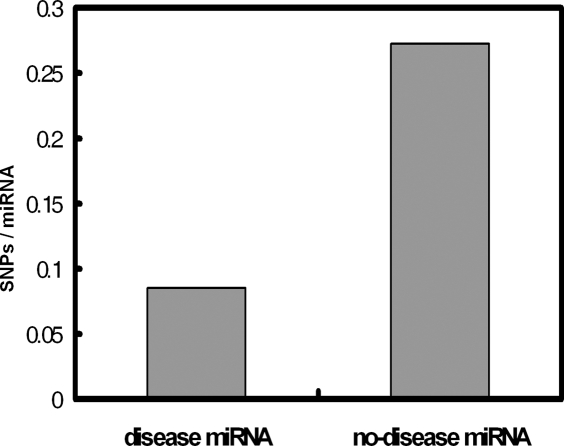
SNP occurring probability of miRNAs implicated in at least one disease (disease miRNA), and miRNAs that are not reported to be implicated in disease (no-disease miRNAs).

**Figure 3:**
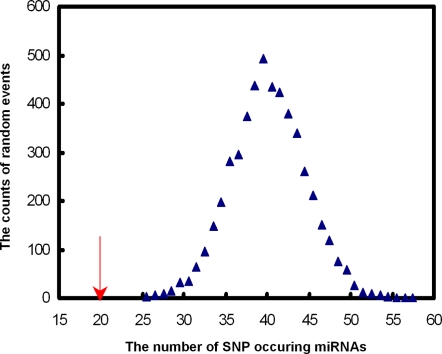
The distribution of the number of SNP occurring miRNAs. The blue triangle indicates the distribution of the number of randomly SNP occurred miRNAs in the miRNAs implicated in at least one disease. The red arrow indicates the real number of SNP occurred miRNAs in the miRNAs implicated in at least one disease.

Single nucleotide polymorphisms (SNPs) are the most common genetic variants in the human genome[20] and SNP density can be used as a metric of the conservation of DNA sequences. We calculated the number of SNPs for miRNAs associated with at least one disease and no diseases. For all 236 miRNAs associated with disease in our dataset, we identified 20 SNPs in 20 miRNAs precursor sequences. The SNP occurring probability is 0.0847 (20/236). The SNP occurring probability of miRNAs that are not associated with diseases is 0.2727 (81/297). Significantly, miRNAs associated with diseases show lower SNP occurring probability than miRNAs that are not associated with diseases (P = 1.3×10^−8^, Fisher's exact test, Figure 2). Similar result was found when searching SNPs in mature miRNA sequences (SNP occurring probability 0.0381 vs. 0.0707, P = 0.07). We also tested the significance by randomly picking up the same number of miRNAs as the number of miRNAs associated with diseases from the whole miRNAs and calculated SNP occurring probability in this group of miRNAs, which was then compared with the real SNP occurring probability. We repeat this process 5000 times and got the P value (P<2.0×10^−4^, Figure 3). These findings suggested that miRNA conservation is associated with human disease susceptibility, which will help in the understanding of miRNAs' roles in diseases.

### miRNA sets and miRNA diseases

The dysfunction of genes in a gene set, such as a signaling pathway or a biological process often result in the same disease. However, it is unclear whether miRNAs implicated in the same disease tend to emerge as miRNA sets. In this study, we defined two types of miRNA sets: miRNA families that contain groups of homologous miRNAs (miRNA duplicates) and miRNA clusters that contain groups of neighboring miRNAs on the human genome.

It has been reported that duplicates of protein coding genes are functionally interchangeable[21], suggesting that they may be involved in similar biological processes with similar roles, therefore, may be implicated in the same disease. However, it is of keen interests but remain unclear whether a family of miRNAs tend to play roles in the same disease. In this study, we revealed that miRNAs in 57% of the diseases have at least one family member in that disease associated miRNAs, which is significantly higher than the random (P = 2.0×10^−4^, Randomization Test). For example, 50% (3/6) of the miRNAs in miR-8 family was related to the thyroid cancer. This finding suggested that the miRNA family members might have similar functions and play roles in similar biological processes, and therefore whose dysfunction would lead to similar phenotype.

*Bartel* and his colleagues reported that neighboring miRNAs show significant coexpression by a microarray profiling analysis[22]. Their finding provided us clues that neighboring miRNAs may be associated with the same disease. To investigate this issue, we identified miRNA clusters that contain neighboring miRNAs and then analyzed the relationship of miRNA clusters and diseases. We found that miRNAs in 46% of the diseases have at least one neighboring member, which is significantly higher than the random (P = 2.0×10^−4^, Randomization Test). For example, all the 6 miRNAs implicated in hematopoietic malignancies are located in the miR-17 cluster. This result indicated that neighboring miRNAs might be regulated by common regulators at similar conditions and function together, and then whose dysfunctions would result in the same disease.

According to the miRNA family and miRNA cluster analysis above, if most members in a miRNA set are associated with one disease, the other members will have a great probability to be associated with that disease too. Therefore, this finding can be used to guide us to predict novel diseases-associated miRNAs.

### Validation in new dataset

All the above analysis was performed on the miRNA-disease association data before November 2007. In order to test the validation of the main results, here we take the miRNA-disease association data between November 2007 and June 2008 to validate the main patterns we found. We first investigated the dysfunctions of miRNAs in similar diseases and different diseases. As a result, 69% (18/26) of the disease pairs in the same class (cancer or other diseases) show similar miRNAs dysfunctions, which is higher than that (33%, 5/15) of the disease pairs between difference disease classes (P = 0.03, Fisher's Exact Test). For example, 5 of the 6 papers revealed a down-regulation of let-7 in cancers, which is consistent with previous report. While all papers reported an up-regulation of let-7 in Alzheimer's disease (see our HMDD database). In the new dataset, miR-195 was reported to be down regulated in human B cell lymphomas[23], which is also consistent with the previous results. We next tested the tissue specificity and disease issue. In our analysis, miR-9 has the highest expression in brain and has a high tissue specificity index (0.63), which is predicted to be associated with brain related diseases. Later research revealed that miR-9 is associated with Alzheimer's disease[24], which supported our prediction. MiR-126 has the highest expression in cardiovascular tissues in our analysis and was reported has high heart specificity[25], and therefore was predicted to be associated with cardiovascular disease and validated by later research[26], in which Harris et al. found miR-126 is associated with vascular inflammation. The SNP occurring probability of miRNAs that are associated with diseases in the new data is 0.0532 (5/94), which is even less than the SNP occurring probability of miRNAs that are associated with diseases in the original data (0.0847). For the miRNA set, we found that miRNAs in 59% (13/22, similar with the original percentage 57%) of the diseases have at least one family member in that disease-associated miRNAs and miRNAs in 45% (10/22, similar with the original percentage 46%) of the diseases have at least one cluster member in that disease-associated miRNAs. These results in the new data suggest the patterns we found are valid and robust.

## Discussion

In a conclusion, we integrated the published human miRNA disease associations and performed a comprehensive analysis to these association data. We uncovered some important patterns between miRNAs and human diseases. These findings will provide help in not only the understanding of human disease and miRNAs but also the identification of novel disease biomarkers at the miRNA level.

Although the study of miRNAs and diseases is an ongoing process and the miRNA-disease association data are far from completeness, our analysis have uncovered statistically significant patterns of miRNA-disease associations. On the other hand, we also noted that some information could not be provided by our analysis. For example, several brain related diseases such as schizophrenia, Parkinson's disease, and neurodegeneration, are not connected to each other in this study. This probably resulted from the incompleteness of the current data. As more comprehensive data becomes available, it will do great help to similar analysis in the future. Another limitation of the current data is that some diseases are not at the same level. For example, B cell lymphoma and mantle cell lymphoma are both belong to lymphoma. Although limitations exist in the current data, the patterns uncovered here is important for understanding the association of miRNAs and various diseases.

## Materials and Methods

### Network analysis

The network is visualized by Pajek (http://vlado.fmf.uni-lj.si/pub/networks/pajek/).

### miRNA expression profile data

We obtained the normalized gene expression profile of 345 miRNAs in 40 normal tissues that included specimens derived from brain, muscle, circulatory, respiratory, lymphoid, gastrointestinal, urinary, reproductive, and endocrine systems[15]. The tissue specificity of a miRNA's expression was calculated based on the tissue specificity index τ proposed by Yanai et al[16]. The τ of miRNA *i* is defined as
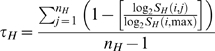
where *n_H_* is the number of human tissues (here *n_H_* = 40) and *S_H_*(*i*,max) is the highest expression value of miRNA *i* across the *n_H_* tissues.

### miRNA SNPs

The human miRNAs are classified into conserved miRNAs and human-specific miRNAs by miRNA family annotations[19]. If a miRNA has other family members in other species, it will be treated as conserved miRNAs; otherwise, it will be treated as human-specific miRNAs. We downloaded human SNP data from dbSNP by using the University of California, Santa Cruz (UCSC) genome browser[27].

### miRNA sets

In this study, we used two types of miRNA sets: families of miRNAs and neighboring miRNAs. We first downloaded all the human miRNAs from miRBase[19]. We next extracted 75 human miRNA families from miRBase (Supplementary Text S2). As *Bartel* et al. reported, 50 kb is an abrupt transition of coexpression between pairs of miRNAs. MiRNAs separated by <50 kb showed high coexpression and miRNAs occurring at a distance of more than 50 kb showed low coexpression[22]. Using this cutoff, we identified 65 miRNA clusters (Supplementary Text S3).

### Statistical computing

All statistical computations were performed in the statistical platform “R”. Randomization test is performed by randomly linking the miRNA-disease associations 5000 times and then calculated the probability of the real case occurs.

## Supporting Information

Figure S1 The bipartite graph model. (A) shows a bipartite graph, which contains two sets of disjoint nodes, here each green node represents one disease and each blue node represents one miRNA and the edges between green nodes and blue nodes represent the associations between miRNAs and diseases. A miRNA-associated disease network (MDN) is constructed if any two diseases share one common associated miRNAs, as shown in (B).
(6.79 MB TIF)

Figure S2 The human miRNA disease network (MDN). Red nodes, green nodes and pink nodes represent cardiovascular diseases, cancers, and other diseases, respectively.
(2.89 MB TIF)

Text S1 miRNAs with high tissue specificity index values
(0.00 MB TXT)

Text S2 miRNA families
(0.00 MB TXT)

Text S3 miRNA clusters
(0.00 MB TXT)
