# Supplementary figures and images for: An Analysis of Human MicroRNA and Disease Associations
Source: PLoS One. 2008 Oct 15;3(10):e3420. doi: 10.1371/journal.pone.0003420 (PMC2559869; doi:10.1371/journal.pone.0003420)

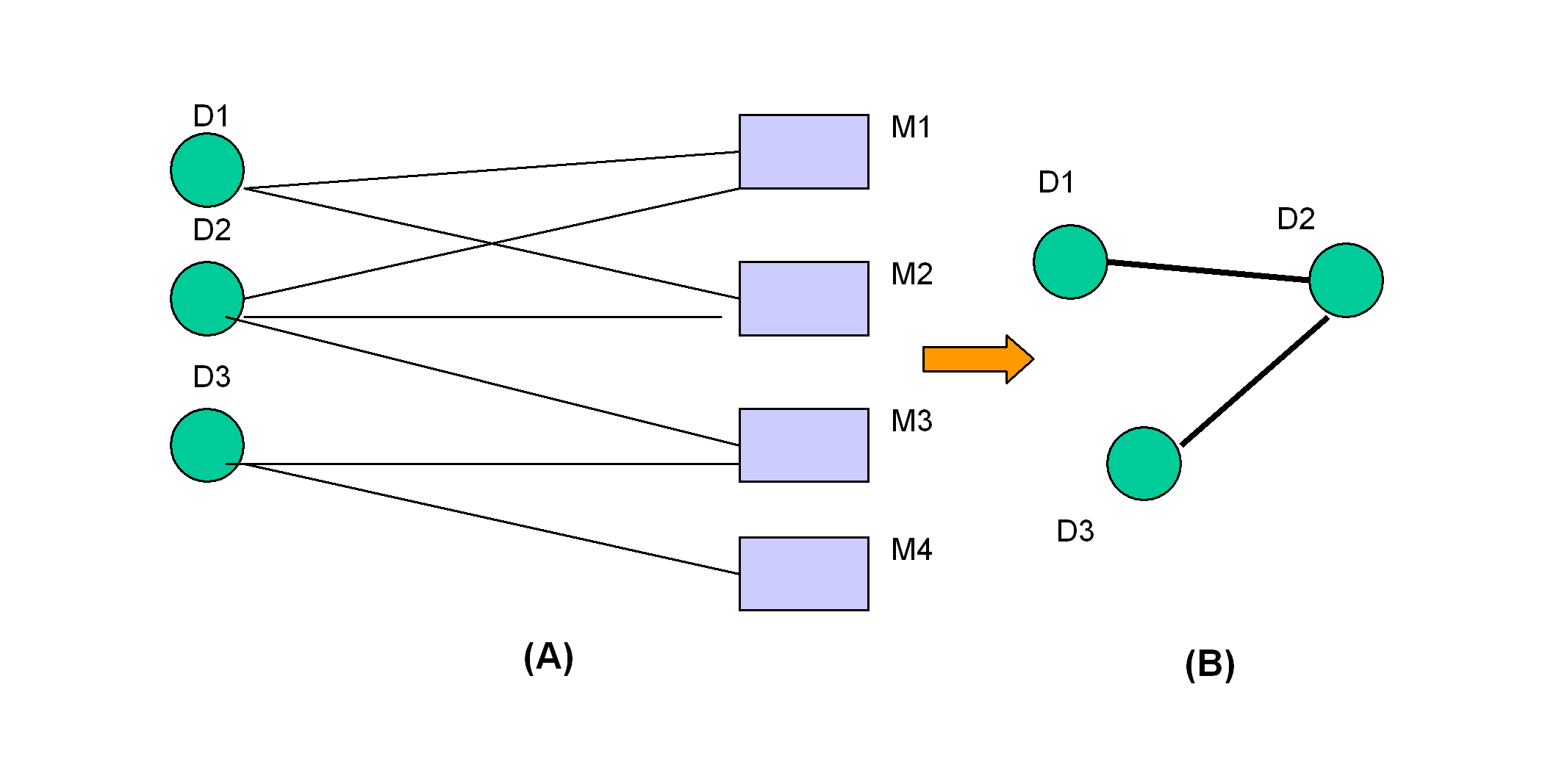

Supplement: Figure S1 — The bipartite graph model. (A) shows a bipartite graph, which contains two sets of disjoint nodes, here each green node represents one disease and each blue node represents one miRNA and the edges between green nodes and blue nodes represent the associations between miRNAs and diseases. A miRNA-associated disease network (MDN) is constructed if any two diseases share one common associated miRNAs, as shown in (B). (6.79 MB TIF) [file pone.0003420.s001.tif]

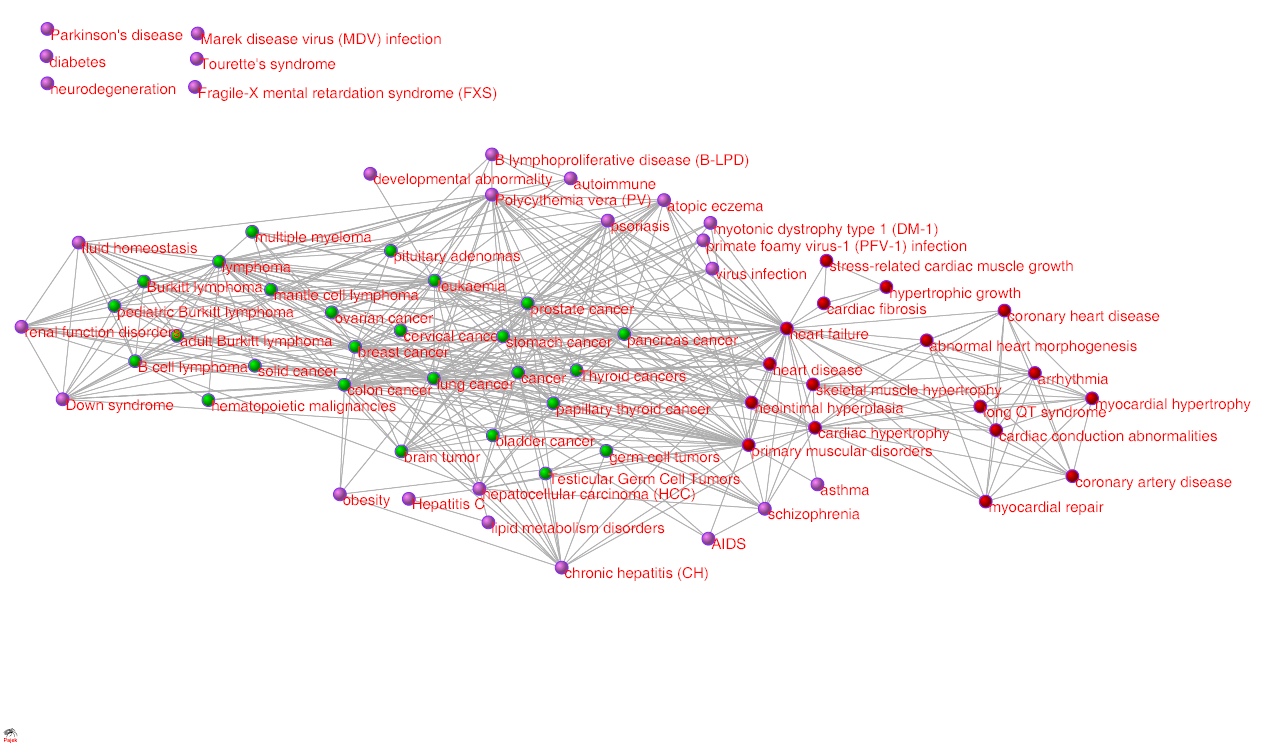

Supplement: Figure S2 — The human miRNA disease network (MDN). Red nodes, green nodes and pink nodes represent cardiovascular diseases, cancers, and other diseases, respectively. (2.89 MB TIF) [file pone.0003420.s002.tif]
